# Mortality Patterns in Patients with Multiple Trauma: A Systematic Review of Autopsy Studies

**DOI:** 10.1371/journal.pone.0148844

**Published:** 2016-02-12

**Authors:** Roman Pfeifer, Michel Teuben, Hagen Andruszkow, Bilal M. Barkatali, Hans-Christoph Pape

**Affiliations:** 1 Department of Orthopedics and Trauma Surgery, Aachen University Medical Center, Aachen, Germany; 2 Department of Trauma and Orthopaedic Surgery, Salford Royal NHS Foundation Trust, Salford, England, United Kingdom; Klinikum rechts der Isar - Technical University Munich - TUM, GERMANY

## Abstract

**Purpose:**

A high percentage (50%-60%) of trauma patients die due to their injuries prior to arrival at the hospital. Studies on preclinical mortality including post-mortem examinations are rare. In this review, we summarized the literature focusing on clinical and preclinical mortality and studies included post-mortem examinations.

**Methods:**

A literature search was conducted using PubMed/Medline database for relevant medical literature in English or German language published within the last four decades (1980–2015). The following MeSH search terms were used in different combinations: “multiple trauma”, “epidemiology”, “mortality “, “cause of death”, and “autopsy”. References from available studies were searched as well.

**Results:**

Marked differences in demographic parameters and injury severity between studies were identified. Moreover, the incidence of penetrating injuries has shown a wide range (between 4% and 38%). Both unimodal and bimodal concepts of trauma mortality have been favored. Studies have shown a wide variation in time intervals used to analyze the distribution of death. Thus, it is difficult to say which distribution is correct.

**Conclusions:**

We have identified variable results indicating bimodal or unimodal death distribution. Further more stundardized studies in this field are needed. We would like to encourage investigators to choose the inclusion criteria more critically and to consider factors affecting the pattern of mortality.

## Introduction

Trauma is still a leading cause of death and has enormous impact on patient’s life and health systems [1]. According to “WHO Global Status Report on Road Safety” more than 1.2 million people die on the world’s roads every year and as many as 50 million others are injured (WHO). Especially in low- and middle income countries road traffic injuries remain a serious health problem [2]. Large national databases allow the identification of risk factors and strategies associated with improved outcome. These databases usually include patients admitted in to the hospital. However, a high rate (50%-60%) of fatally injured victims do not arrive at hospital and die due to trauma prior to arrival [3]. Preclinical mortality studies and investigations of causes of deaths proven by an autopsy have not been frequently performed. However, this data can provide valuable information to identify clinical problems.

The first publication and statistical analysis done over 30 years ago related to preclinical and clinical trauma has observed a trimodal pattern of mortality [4]. Moreover, this autopsy based study allowed the identification of the immediate causes of death and injury patterns. Other autopsy studies have provided important information regarding injury severity, distribution of organ damage, the importance of missed injuries and possible preventability of deaths [5,6]. Despite its value, the autopsy rate in most countries remains low. In order to understand the patterns and trends in mortality after trauma over the last decades, we have performed a review of literature focusing on clinical and preclinical mortality including post-mortem examinations.

## Materials and Methods

A review of the literature was performed on available publications including autopsy data in severely injured patients. A literature search was conducted using PubMed/Medline database for relevant medical literature in English or German language published within the last four decades (1980–2015). The following MeSH search terms were used in different combinations: “*multiple trauma*”, “*epidemiology*”, “*mortality”*, “*causes of death*”, and “*autopsy*”. Further searches employed combination of terms and synonyms. References of retrieved publications were also searched in order to identify additional papers that may have been missed via the electronic search.

Studies were included if they fit to the following inclusion criteria:

- Original research papers published between January 1, 1980 and September 31, 2015
- Published in a peer-review journals of any study design (prospective and retrospective)
- Focus on blunt and penetrating trauma
- Autopsy of trauma patients performed
- No review articles, textbook chapters, posters and abstracts

Publications were chosen if they contained adequate information (at least two parameters for tables). Literature was chronologically summarized in tables. Variables of interest were: age of patients, gender (percentage of male), injury severity described by Injury Severity Score (ISS), pattern of mortality (unimodal, bimodal, etc.). type of trauma (blunt versus penetrating), and causes of death determined by an autopsy.

## Results

**Fig 1:**
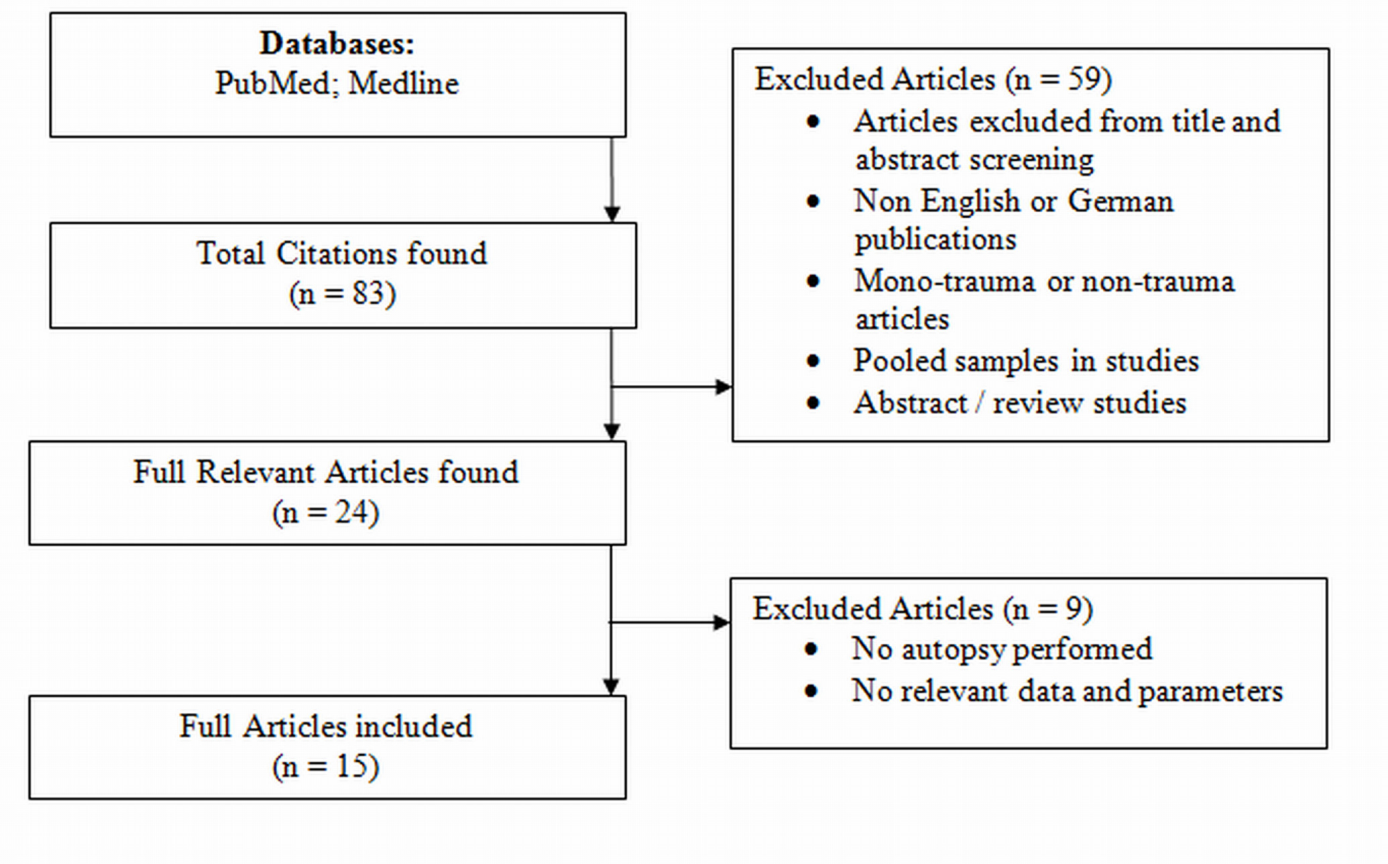
This flow diagram demonstrates the inclusion and exclusion of articles found in databases.

The standardized search revealed a total of 15 publications: There were 3 reports published between 1980 and 1989; 3 reports between 1990 and 1999; 7 articles between 2000 and 2010, and 2 publications between 2011 and 2015. Fig 1 shows a flow diagram of studies included and excluded according to the eligibility criteria. The initial search revealed 83 relevant publications, of which 68 were excluded (reviews, abstracts, mono-trauma, no autopsy, etc.). The remaining 15 publications were included in to the final analysis. Table 1 describes the inclusion and exclusion criteria of studies reviewed. Almost all reports from the year 2000 to 2015 included a detailed list of inclusion and exclusion criteria. The majority of studies excluded non-traumatic deaths such as: burns, hanging, drowning, etc. In articles published prior the year 2000 these data were only mentioned in minority of studies.

**Table 1:** Summary of inclusion and exclusion criteria of studies included in to this review.

References	Pat. (No)	Inclusion Criteria	Exclusion Criteria
(Baker et al., 1980)[7]	437	all accidental trauma	ND
(Pories et al., 1989)[19]	54	all trauma victims	ND
(Shackford et al., 1989)[20]	104	all major trauma victims	ND
(Sahdev et al., 1994)[9]	177	all road traffic fatalities	ND
(Sauaia et al., 1995)[18]	289	all trauma deaths	ND
(Meislin et al., 1997)[17]	710	all trauma deaths	drowning, poisoning, burns
		(<18 month after trauma)	overdose
(Hodgson et al., 2000)[21]	108	all blunt trauma deaths	ND
(Marson et al., 2001)[22]	115	motor vehicle crash deaths	ND
(Chiara et al., 2002)[14]	255	all trauma deaths	poisoning, drowning, overdose
			hanging
(Stewart et al., 2003)[23]	753	all trauma deaths	ND
(Tien et al., 2007)[24]	558	all trauma deaths	burns, drowning, hanging
(Søreide et al., 2007)[11]	260	all trauma deaths from	non-residents, hanging,
		study area	drowning, poisoning, suffocation
(Pang et al., 2008)[25]	186	all trauma deaths	non-residents
(Evans et al.,2010)[15]	175	all trauma deaths	electrocution, drowning, hanging
			strangling, poisoning
(Kleber et al. 2012) [26]	440	all trauma deaths	strangulation, burns, drowning,
			Non-traumatic deaths

ND = no data

Table 2 demonstrated the demographic parameters and the injury severity score described in reviewed studies. Over the observation period of 35 years we have found an increase of mean age of included study populations. The rate of male trauma patients was reported between 55% and 88%. Moreover, the injury severity has been shown to have a wide range between the studies. The lowest ISS value was 9 and the highest was reported with 62.3.

**Table 2:** Demographic parameters of patients included in to the study. Over the observation period of 30 years we observed an increased age of study populations. The meant Injury Severity Score (ISS) has shown a wide range between 9 points and up to 62.3 points.

References	Age (Years)	Gender (% of Male)	ISS (Points)
(Baker et al., 1980)[7]	65%<51	75.3	mean 43
(Pories et al., 1989)[19]	median 28	74	mean 9
(Shackford et al., 1989)[20]	mean 34.5	ND	mean 49.5
(Sahdev et al., 1994)[9]	mean 35	88	mean 37.8
(Sauaia et al., 1995)[18]	mean 36.8	79	mean 35.7
(Meislin et al., 1997)[17]	mean42.9–49.3	79.6–67.9	Mean 39–25.6
(Hodgson et al., 2000)[21]	median 39	72	mean 30–43
(Marson et al., 2001)[22]	mean 33.9–35.3	81.3–82.8	ND
(Chiara et al., 2002)[14]	mean 44–55	73	mean 27.5–62.3
(Stewart et al., 2003)[23]	mean 42.5	ND	mean 41.2
(Tien et al., 2007)[24]	mean 48.7	71.2	mean 38.8
(Søreide et al., 2007)[11]	mean 45.8	75	median 38
(Pang et al., 2008)[25]	mean 36.5	74	median 25
(Evans et al.,2010)[15]	mean 55	55	mean 36
(Kleber et al. 2012) [26]	mean 58	64.1	ND

ND = no data

Table 3 summarizes the rate of penetrating and blunt trauma. All publications distinguished between these two trauma entities. The majority of studies (12 out of 15 papers) included both penetrating and blunt trauma victims in to their studies. The rate of penetrating injuries has also shown a wide range from 4% to 38%. The majority of the studies focused on preclinical and clinical patients. Four out of 15 publications did not include preclinical deaths.

**Table 3:** The majority of studies included both penetrating and blunt trauma patients. The rate of penetrating injuries shows a wide range from 4% to 38%. However, 4 studies excluded preclinical deaths from the analysis.

References	Penetrating Trauma (%)	Blunt Trauma (%)	Pre-clinical Deaths (Yes)
(Baker et al., 1980)[7]	31	45.7	X
(Pories et al., 1989)[19]	13	84	-
(Shackford et al., 1989)[20]	37.5	57.7	-
(Sahdev et al., 1994)[9]	-	100	X
(Sauaia et al., 1995)[18]	49	48	X
(Meislin et al., 1997)[17]	Up to35	34–71	X
(Hodgson et al., 2000)[21]	-	100	-
(Marson et al., 2001)[22]	-	100	X
(Chiara et al., 2002)[14]	22	78.1	X
(Stewart et al., 2003)[23]	21	71	-
(Sharma et al., 2005)[27]	up to 6.3	56.9	X
(Tien et al., 2007)[24]	13	80	X
(Søreide et al., 2007)[11]	13	87	X
(Pang et al., 2008)[25]	4.8	53.2	X
(Evans et al., 2010)[15]	14	76	X
(Kleber et al., 2012)[26]	13.2	78.6	X

Table 4 gives an overview over patterns of mortality and most frequent causes of death. Our review has identified 5 studies with a unimodal distribution of mortality. In these studies, only one peak (mostly preclinical or direct after admission) was observed with a homogenous post-traumatic course. In the other hand 4 publications have described a bimodal pattern. These studies identified a further peak during a clinical course mostly related to late deaths. Four peaks were reported by one study. One work has identified a model-dependent relation between the pattern of death and time intervals chosen. Almost all studies (12 out of 15) have shown consistent results regarding the causes of death. Brain injury was still the leading cause of death over the period of 30 years. Exsanguination (10 out of 15 studies) and combination of brain injury and hemorrhagic shock was reported in 6 out of 15 studies.

**Table 4:** Pattern of mortality and three most frequent causes of death over the period of 30 years.

References	Pattern of mortality	Caused of deaths
(Baker et al., 1980)[7]	ND	BI ; TI ; HS
(Pories et al., 1989)[19]	ND	BI, HS ; Other
(Shackford et al., 1989)[20]	ND	BI ; HS ; TI
(Sahdev et al., 1994)[9]	Four peaks	BI, HS ; BI+HS
(Sauaia et al., 1995)[18]	Bimodal	BI ; HS ; MOF
(Meislin et al., 1997)[17]	Bimodal	BI ; HS ; Other
(Hodgson et al., 2000)[21]	Bimodal	BI ; Sepsis ; HS
(Marson et al., 2001)[22]	Unimodal	BI; HS; BI+HS
(Chiara et al., 2002)[14]	Unimodal	BI+HS ; HS ; BI
(Stewart et al., 2003)[23]	Unimodal	BI ; HS ; BI+HS
(Tien et al., 2007)[24]	ND	BI ; HS ; BI+HS
(Søreide et al., 2007)[11]	Model-dependent	BI ; HS ; MOF
(Pang et al., 2008)[25]	Unimodal	BI; HS; BI+HS
(Evans et al., 2010)[15]	Unimodal	BI; HS; BI+HS
(Kleber et al. 2012)[26]	Bimodal	PT; BI; HS

A trimodal distribution has not been confirmed in autopsy studies. Unimodal and bimodal distribution of deaths has ben described. Moreover, brain injury, exsanguination and combination of brain injury and severe bleeding were the leading causes of death after trauma.

ND = No Data; BI = Brain Injury; TI = Thoracic Injury; HS = Haemorrhagic Shock; MOF = Multiple Organ Failure; PT = Polytrauma

## Discussion

The analysis of the distribution of trauma deaths is crucial to identify areas of improvement in pre-clinical and hospital trauma care. A trimodal distribution of deaths was described over three decades ago and is still considered as standard in most textbooks and literature [4,7,8]. The first peak was classified as immediate death (with in 60 minutes) and included up to 45% of all trauma victims. The main cause of death in these patients was major injury of central nervous system or cardiovascular injuries. The second peak included early deaths (within 1 to 4 hours) and accounted for about 34% of death. Cardiovascular and neurological injuries were also the predominant causes of death. The third peak, which was considered the lowest (20%), included late deaths (> 1 week). Most of these patients died due to systemic complications, such as sepsis or multiple organ failure (MOF) [4].

Our literature review revealed that papers published in the last two decades did not confirm the trimodal distribution summarized above. Several investigations have reported unimodal and bimodal distributions of mortality regardless of geographical location with relevant differences in the presence of early and late deaths (Table 3). An Indian study published by Sahdev et al. reported an additional fourth peak between day one and two and classified as “delayed deaths” [9]. One reason for this additional peak might be the differences between present trauma systems at the time point of analysis. Demetriades et al. have shown in a large study of trauma deaths that there is no specific universal temporal distribution of death [10]. The authors critically discussed that the distribution of death highly depends on mechanisms of injury, the injury patterns, injury severity and age of the patients. Moreover, this analysis has clearly shown that the temporal distribution of death in penetrating trauma is different from that in blunt trauma [10]. Patients with penetrating injuries died more frequently within the first 60 minutes (immediate death). While blunt trauma patients rarely died within the first hour or on scene [10]. Søreide et al. also questioned the trimodal distribution and postulated that the temporal distribution is an effect of selected time interval model [11]. The authors noted that temporal distribution merely serves as an educational tool. Data from two large European registries (Trauma Registry of German Society of Trauma Surgery and Trauma Audit and Research Network (TARN) UK) was used to characterize trauma deaths according to the time point of death [12]. These large databases confirm that the time to death after severe trauma does not follow the trimodal distribution, but rather show a consistently decreasing mortality rate over time [12].

Brain injury is still the leading cause of death after multiple trauma. Especially early deaths (up to 24 hours) and first week deaths are more often due to brain injury [13]. The second frequent cause of death is exsanguination mainly due to thoracic or abdominal injuries. Isolated hemorrhage is predominant in penetrating trauma, while brain injuries with combination with severe bleeding were more frequently observed with blunt trauma [14]. Focusing on late post-traumatic deaths Søreide and coauthors observed that patients who died due to Multiple Organ Failure (MOF) were predominantly older and less severely injured in comparison to those who died early [11]. Whether the initial trauma deaths are preventable or not, has been also discussed in studies. Chiara and co-authors considered patients with at least one sustained injury with AIS of 6 or head injury with ≥5 as unsalvageable. Other studies analyzed trauma death in the first hour and calculated a potential salvageable death rate of up to 7% [5,14].

The advantage of post-mortem examinations after trauma is the exact identification of injury severity, injury patterns and causes of death. However, the interpretation of this data is complex and difficult. In severely injured blunt trauma patients fatal injuries may affect multiple different organ systems and body areas. The differentiation whether severe brain injury or thoracic injury with heart or vessel lesions is the primary cause of death is complex. In this case the definition of leading injury according to MAIS might be useful. It is an objective and standardized way to graduate the severity of injuries present after trauma. However, only a few studies documented and classified the organ injuries according to AIS.

We have identified great variability between the studies. Especially in the inclusion criteria used by the different publications reviewed. Most studies included both penetrating and blunt trauma patients (Table 2). The percentage of patients with penetrating trauma ranged between 4% and 38%. As previously described, penetrating trauma has been shown to have a different pattern of mortality with predominantly higher rates of immediate and early deaths. Thus, discrepancies in penetrating and blunt trauma rates make the studies less comparable. Moreover, trauma mechanisms, such as suicides, hanging, or burns may also have an effect on mortality pattern, but also have been included in several analyses. Secondly, in order to calculate the temporal distribution, studies have shown a wide heterogeneity of time intervals chosen. This discrepancy may also be responsible for inconsistency reported by authors. Previous studies have encouraged standardized reporting of data after trauma [11]. Thirdly, the demographic parameters have also shown a wide range (Table 1). Only a few studies documented a representativity of the included study population. According to previous studies, age is a relevant factor and affects the distribution [10,15]. Elderly trauma victims more frequently sustain low energy trauma. The presence of comorbidities and medications appears to increase the risk of developing systemic complications during the clinical course [16]. Finally, different trauma system are known to influence both time and location of trauma deaths [16]. Geographical diversity might be one explanation for differences in pre-clinical mortality. Several investigators excluded the preclinical death in early studies; and therefore missed relevant pre-clinical patient data collection. Other investigations have demonstrated that the implementation of a new trauma system has led to modifications of temporal pattern of mortality [17,18]. We suggest that a short paragraph is included describing the trauma system in future studies, in order to consider the effects of the different trauma systems.

## Conclusion

According to our review, there appears to be a consensus in that the temporal distribution of trauma deaths is uni- or bimodal. It is difficult to differentiate whether the pattern of mortality has changed due to improvement of trauma care or due to differences in reviewed studies. We have identified clear variability between publications. We feel that further more standardized studies in this field are required. We would like to encourage investigators to choose the inclusion criteria more critically, to compare their demographic data to the geographic population, and to consider factors affecting the pattern of mortality. Moreover, autopsy studies have a huge value since they allow the identification of exact causes of deaths and injury patterns. Brain Injuries followed by exsanguination are still leading causes of death. However, the interpretation of data is not always easy. (How severe were these injuries? Which injury was lethal? Which combinations are deadly?). Standardized and objective documentation of lesions and injuries (e.g. AIS or others) is of importance and allows further studies to compare their results. Moreover, we feel that future studies should separately consider the impact of penetrating versus blunt injuries and the role of concomitant injuries.

## Supporting Information

S1 PRISMA Checklist PRISMA Checklist. (DOC)

## Abbreviations

ATLS: Anvanced Trauma Life Support
AIS: Abbreviated Injury Scale
ISS: Injury Severity Score
MOF: Multiple Organ Failure
TARN: Trauma Audit and Research Network

## References

[1] World Health Organization. Global burden of disease, 2004 update Geneva: WHO Press; 2008.

[2] World Health Organization. Global status report on road safety 2013: supporting a decade of action Geneva: WHO Press; 2013.

[3] Pfeifer R, Tarkin IS, Rocos B, Pape HC. Patterns of mortality and causes of death in polytrauma patients—has anything changed? Injury 2009 9;40(9):907–11. 10.1016/j.injury.2009.05.006 19540488

[4] Trunkey DD, Lim RC. Analysis of 425 Consecutive Trauma Fatalities. J Am Coll Emerg Phys 1974;Nov-Dec:368–71.

[5] MacLeod JB, Cohn SM, Johnson EW, McKenney MG. Trauma deaths in the first hour: are they all unsalvageable injuries? Am J Surg 2007 2;193(2):195–9. 17236846 10.1016/j.amjsurg.2006.09.010

[6] Ong AW, Cohn SM, Cohn KA, Jaramillo DH, Parbhu R, McKenney MG, et al Unexpected findings in trauma patients dying in the intensive care unit: results of 153 consecutive autopsies. J Am Coll Surg 2002 4;194(4):401–6. 11949744 10.1016/s1072-7515(02)01123-7

[7] Baker CC, Oppenheimer L, Stephens B, Trunkey DD. Epidemiology of Trauma Deaths. Am J Surg 1980;140:144–8. 7396078 10.1016/0002-9610(80)90431-6

[8] Trunkey DD. Trauma. Sci Am 1983;249:28–53. 6623052

[9] Sahdev P, Lacqua MJ, Singh B, Dogra TD. Road Traffic Fatalities in Delhi: Causes, Injury Patterns, and Incidence of Preventable Deaths. Accid Anal and Prev 1994;26(3):377–84. 10.1016/0001-4575(94)90011-6 8011050

[10] Demetriades D, Murray J, Charalambides K, Alo K, Velmahos G, Rhee P, et al Trauma Fatalities: Time and Location of Hospital Deaths. J Am Coll Surg 2004;198(1):20–9. 14698307 10.1016/j.jamcollsurg.2003.09.003

[11] Søreide K, Krüger AJ, Vardal AL, Ellingsen CL, Soreide E, Lossius HM. Epidemiology and Contemporary Patterns of Trauma Deaths: Changing Place, Similar Pace, Older Face. World J Surg 2007;31:2092–103. 17899256 10.1007/s00268-007-9226-9

[12] Lefering R, Paffrath T, Bouamra O, Coats TJ, Woodford M, Jenks T, et al Epidemiology of in-hospital deaths. Eur J Trauma Emerg Surg 2012;38:3–9. 10.1007/s00068-011-0168-4 26815666

[13] Goris RJ, Draaisma J. Causes of death after blunt trauma. J Trauma 1982;22(2):141–6. 7062358 10.1097/00005373-198202000-00011

[14] Chiara O, Scott JD, Cimbanassi S, Marini A, Zoia R, Rodriguez A, et al Trauma deaths in an Italian urban area: an audit of pre-hospital and in-hospital trauma care. Injury 2002;33:553–62. 12208056 10.1016/s0020-1383(02)00123-7

[15] Evans JA, van Wessem KJ, McDougall D, Lee KA, Lyons T, Balogh ZJ. Epidemiology of traumatic deaths: comprehensive population-based assessment. World J Surg 2010 1;34(1):158–63. 10.1007/s00268-009-0266-1 19882185

[16] Bamvita JM, Bergeron E, Lavoie A, Ratte S, Clas D. The Impact of Premorbid Conditions on Temporal Pattern and Location of Adult Blunt Trauma Hospital Deaths. J Trauma 2007;63:135–41. 17622881 10.1097/TA.0b013e318068651d

[17] Meislin H, Criss EA, Judkins D, Berger R, Conroy C, Parks B, et al Fatal Trauma: The Modal Distribution of Time to Death Is a Function of Patient Demographics and Regional Resources. J Trauma 1997;43(3):433–40. 9314304 10.1097/00005373-199709000-00008

[18] Sauaia A, Moor FA, Moore EE, Moser KS, Brennan R, Read RA, et al Epidemiology of Trauma Deaths: A Reassessment. J Trauma 1995;38(2):185–93. 7869433 10.1097/00005373-199502000-00006

[19] Pories SE, Gamelli RL, Pilcher DB, Vacek P, Jones J, Harris F, et al Practical Evaluation of Trauma Deaths. J Trauma 1989;29(12):1607–10. 2593185 10.1097/00005373-198912000-00001

[20] Shackford SR, Mackersie RC, Davis JW, Wolf PL, Hoyt DB. Epidemiology and Pathology of Traumatic Deaths Occuring at a Level 1 Trauma Center in a Regionalized System: The Importance of Secondary Brain Injury. J Trauma 1989;29(10):1392–7. 2810417 10.1097/00005373-198910000-00018

[21] Hodgson NF, Stewart TC, Girotti MJ. Autopsies and death certification in deaths due to blunt trauma: what are we missing? Can J Surg 2000;43(2):130–6. 10812348 PMC3695126

[22] Marson AC, Thomson JC. The Influence of Prehospital Trauma Care on Motor Vehicle Crash Mortality. J Trauma 2001;50:917–21. 11371852 10.1097/00005373-200105000-00024

[23] Stewart RM, Myers JG, Dent DL, Ermis P, Gray GA, Villarreal R, et al Seven Hundred Fifty-Three Consecutive Death in a Level 1 Trauma Center: The Argument for Injury Prevention. J Trauma 2003;54:66–71. 12544901 10.1097/00005373-200301000-00009

[24] Tien HC, Spencer F, Tremblay LN, Rizoli SB, Brenneman FD. Preventable Death From Hemorrhage at a Level I Canadian Trauma Center. J Trauma 2007;62:142–6. 17215745 10.1097/01.ta.0000251558.38388.47

[25] Pang JM, Civil I, Ng A, Adams D, Koelmeyer T. Is the Trimodal Pattern of Death after Trauma a dated Conceptin the 21st Century? Trauma deaths in Auckland 2004. Injury 2008;39:102–6. 17880967 10.1016/j.injury.2007.05.022

[26] Kleber C, Giesecke MT, Tsokos M, Haas NP, Schaser KD, Stefan P, et al Overall distribution of trauma-related deaths in Berlin 2010: advancement or stagnation of German trauma management? World J Surg 2012 Sep;36(9):2125–30. 10.1007/s00268-012-1650-9 22610265

[27] Sharma BR, Harish MGD, Singh VP. Missed diagnoses in trauma patients vis-a`-vis significance of autopsy. Injury 2005;36:976–83. 16005004 10.1016/j.injury.2004.09.025

